# Phase angle as a prognostic nutritional marker for survival in nasopharyngeal carcinoma patients undergoing chemoradiotherapy: a prospective cohort study

**DOI:** 10.3389/fnut.2026.1830829

**Published:** 2026-06-15

**Authors:** Zhang Yanxin, Jiang Jiaping, Liang Limin, Huang Xiaojun, Yang Guirong, Li Wei, Tang Qiuhong, Wei Lina, Chen Xin, Lu Jiamei

**Affiliations:** Department of Radiotherapy, The First Affiliated Hospital of Guangxi Medical University, Nanning, Guangxi, China

**Keywords:** nasopharyngeal carcinoma phase angle, nutritional marker, chemoradiotherapy, prognosis, survival

## Abstract

**Background:**

Conventional nutritional indicators have limitations in predicting outcomes for nasopharyngeal carcinoma (NPC) patients undergoing chemoradiotherapy. Phase angle (PA), a bioelectrical impedance-derived measure of cellular integrity, has emerged as a prognostic marker in various malignancies but remains understudied in NPC. This study investigated whether PA provides superior prognostic information for overall survival (OS) compared to conventional indicators.

**Methods:**

In this prospective cohort study, 380 newly diagnosed NPC patients scheduled for chemoradiotherapy were enrolled. PA was measured using InBody S10. Nutritional status was assessed via body mass index (BMI), serum albumin, and the Patient-Generated Subjective Global Assessment (PG-SGA). OS was the primary endpoint. For Kaplan–Meier visualization, patients were stratified by the median PA value. Multivariable Cox regression and time-dependent ROC analyses were performed.

**Results:**

Over a median 60-month follow-up, 27 deaths (7.1%) occurred. PA showed significant correlations with BMI (r = 0.512, *p* < 0.001), albumin (r = 0.685, p < 0.001), and PG-SGA (r = −0.738, p < 0.001). After adjustment for age and TNM stage, PA independently predicted OS (HR = 0.638 per 1°increase, 95% CI: 0.483–0.853, *p* = 0.002). PA demonstrated promising predictive accuracy (AUC = 0.742, 95% CI: 0.655–0.829). Given the moderate event number, formal AUC comparisons were not performed.

**Conclusion:**

PA may be an independent prognostic factor for newly diagnosed NPC patients undergoing chemoradiotherapy. These findings may support its potential utility in baseline risk assessment, though validation in larger cohorts is warranted.

## 1 Introduction

Nasopharyngeal carcinoma (NPC) exhibits a striking geographic distribution, with a notably high incidence in China—approximately 20 cases per 100,000 population—compared to fewer than 1 case per 100,000 in Europe and North America (1). Intensity-modulated radiotherapy (IMRT)-based concurrent chemoradiotherapy serves as the cornerstone of treatment for nasopharyngeal carcinoma (2). However, it frequently induces oral and oropharyngeal mucositis, resulting in pain that compromises normal food intake. Moreover, chemotherapy-induced nausea and vomiting further exacerbate nutritional deterioration, collectively contributing to the development of malnutrition. Literature reports indicate that before treatment for nasopharyngeal carcinoma, 8.7–10.3% of patients present with malnutrition. During treatment, however, the proportion of patients experiencing weight loss exceeding 5% rises substantially, ranging from 53.6 to 70.2% (3, 4). Malnutrition not only compromises patient sensitivity to chemoradiotherapy but also contributes to prolonged hospitalization, increased medical costs, diminished quality of life, and even mortality attributable to malnutrition and its associated complications (5, 6).

Therefore, identifying early and effective indicators for malnutrition risk and prognostic assessment may provide valuable guidance for optimizing personalized nutritional support in cancer patients. Current clinical practice still relies on conventional nutritional indicators, including body mass index (BMI), serum albumin, and the Patient-Generated Subjective Global Assessment (PG-SGA) (7–9). Despite their widespread clinical use, these conventional indicators are subject to inherent limitations that constrain their predictive value, underscoring the need for more reliable prognostic markers (10). Body mass index (BMI), for example, fails to distinguish between adipose tissue and lean body mass, and its interpretability is further confounded by fluid retention—a common clinical manifestation in cancer patients (8). Serum albumin, despite its ease of measurement, is confounded by systemic inflammation and hepatic synthetic function, which undermines its specificity as a pure nutritional biomarker (9). The Patient-Generated Subjective Global Assessment (PG-SGA), despite its comprehensiveness, is inherently subjective and time-consuming, which limits its routine applicability in busy clinical settings (7). Collectively, these limitations underscore the need for a more objective, reproducible, and prognostically robust nutritional indicator.

Bioelectrical impedance analysis (BIA) is a simple, cost-effective, and non-invasive method widely used for body composition assessment in patients with cancer. By measuring whole-body impedance and exploiting the electrical properties of tissues, BIA enables the evaluation of cellular function, hydration status, and nutritional condition. Among the parameters derived from BIA, phase angle (PA) has emerged as a key indicator (11). PA serves as an objective indicator reflecting the structural integrity and functional status of the cell membrane. The cell membrane is composed of a phospholipid bilayer, in which lipids play an essential role in maintaining its structural and functional integrity (12, 13). A reduced phase angle reflects tumor-induced metabolic depletion, systemic inflammation, and derangements in body composition, manifesting as protein-energy wasting, diminished cellular membrane capacitance, and an expanded extracellular water compartment (14). Accumulating evidence has substantiated the prognostic value of phase angle across diverse malignancies (15), including pancreatic (16), lung (17), and breast cancers, wherein lower phase angle consistently predicts poorer survival.

However, despite this growing body of evidence, its prognostic value in NPC remains conspicuously understudied. Critically, no prospective study to date has systematically compared its predictive performance against the panel of conventional nutritional indices routinely employed in NPC management. Whether phase angle offers incremental or superior prognostic information beyond these established markers in the specific context of NPC patients undergoing chemoradiotherapy is a question that has yet to be definitively addressed.

Given the existing research gaps, this prospective cohort study was designed with two primary objectives. The first was to evaluate the prognostic predictive value of the phase angle in newly diagnosed nasopharyngeal carcinoma patients undergoing chemoradiotherapy. The second was to compare the phase angle with conventional nutritional indicators, including body mass index, serum albumin and Patient-Generated Subjective Global Assessment (PG-SGA), and to systematically compare their predictive efficacy for overall survival.

## 2 Materials and methods

### 2.1 Study design and population

This prospective cohort study enrolled patients with newly diagnosed, histologically confirmed nasopharyngeal carcinoma (NPC) admitted between April 2021 and April 2026, with follow-up data collected until April 2026, resulting in a median follow-up of 60 months. Patients with recurrent or metastatic disease at presentation were excluded to ensure population homogeneity regarding treatment intent and prognosis. The study protocol was approved by the Institutional Ethics Committee of The First Affiliated Hospital of Guangxi Medical University (Approval No. 2023-S913-01), and written informed consent was obtained from all participants before enrollment.

A total of 395 consecutive patients with newly diagnosed NPC scheduled to receive chemoradiotherapy were initially screened for eligibility. Of these, 15 were excluded for the following reasons: five did not meet the inclusion criteria (three aged >75 years, two with ECOG performance status >2); four declined participation; three had implantable electronic devices interfering with bioelectrical impedance analysis; two had clinically significant edema or ascites; and one had a history of other malignancy within 5 years. Consequently, a total of 380 patients were prospectively enrolled in the final cohort.

#### 2.1.1 Inclusion criteria

Patients were eligible for inclusion if they met all of the following criteria:

(1) Histologically confirmed newly diagnosed nasopharyngeal carcinoma;
(2) Planned to receive definitive chemoradiotherapy (concurrent chemoradiotherapy with or without induction chemotherapy);
(3) Aged 18–75 years;
(4) Eastern Cooperative Oncology Group (ECOG) performance status of 0–2;
(5) Life expectancy of at least 6 months.

#### 2.1.2 Exclusion criteria

Patients were excluded if they met any of the following criteria:

(1) Presence of implantable electronic devices (e.g., cardiac pacemakers) or metal implants that could interfere with bioelectrical impedance analysis (BIA) measurements.
(2) Clinically significant edema, ascites, or pleural effusion, which may confound BIA-derived parameters
(3) History of other malignancies within the past 5 years.
(4) Severe hepatic dysfunction (Child–Pugh class C) or severe renal impairment (estimated glomerular filtration rate <30 mL/min/1.73 m^2^).
(5) Pregnancy or lactation.
(6) Inability or unwillingness to comply with the scheduled follow-up assessments.

### 2.2 Treatment regimens

All patients were newly diagnosed and received definitive intensity-modulated radiotherapy (IMRT) as the foundational modality. Concurrent chemoradiotherapy (CCRT) with cisplatin (100 mg/m^2^ on days 1, 22, and 43 of radiotherapy) was administered as the standard regimen. For patients with locoregionally advanced disease, induction chemotherapy (IC) followed by CCRT was administered at the discretion of the treating physician. Induction chemotherapy regimens included GP (gemcitabine + cisplatin), TP (paclitaxel + cisplatin), or TPF (docetaxel + cisplatin + fluorouracil).

### 2.3 Data collection and measurements

#### 2.3.1 Baseline characteristics

At enrollment, comprehensive demographic and clinical data were systematically collected for each participant, including age, sex, smoking status, and alcohol consumption. For newly diagnosed patients, tumor stage according to the 8th edition of the American Joint Committee on Cancer/Union for International Cancer Control (AJCC/UICC) staging system. Treatment-related information included the intended treatment modality (concurrent chemoradiotherapy alone vs. induction chemotherapy followed by concurrent chemoradiotherapy). Anthropometric measurements comprised height, weight, and calculated body mass index (BMI). Nutritional evaluation was performed using the Patient-Generated Subjective Global Assessment (PG-SGA) by trained clinical dietitians within 72 h of enrollment.

#### 2.3.2 Bioelectrical impedance analysis and phase angle measurement

Bioelectrical impedance analysis (BIA) was performed using a multi-frequency segmental body composition analyzer (InBody S10, Biospace Co., Ltd., Seoul, Republic of Korea). This device employs eight tactile electrodes (four in contact with each hand and each foot) and operates at multiple frequencies (1, 5, 50, 250, 500, and 1,000 kHz), enabling precise measurement of body composition parameters, including phase angle.

To ensure measurement reproducibility and minimize inter-individual variability, all assessments were conducted under strictly standardized conditions: following an overnight fast of at least 8 h, with an empty bladder, after a minimum of 10 min of rest in the supine position, at a controlled ambient temperature of 20–25 °C. During measurement, patients were instructed to remain motionless and refrain from speaking to avoid interference with impedance signals. PA at 50 kHz was directly recorded from the BIA device, as this frequency is most commonly reported in oncological studies and provides optimal sensitivity for cellular health assessment. The intra-observer coefficient of variation for PA measurements at our institution was less than 2%, indicating excellent reliability.

#### 2.3.3 Laboratory assessments

Fasting venous blood samples were collected by trained nurses under strict aseptic conditions between 6:00 and 7:00 a.m. following an overnight fast. All analyses were performed by dedicated laboratory technicians who were blinded to clinical data.

#### 2.3.4 Conventional nutritional indicators

Body mass index (BMI) was calculated as weight in kilograms divided by the square of height in meters (kg/m^2^). Serum albumin concentrations were obtained from the laboratory assessments described in Section 2.3.3. The Patient-Generated Subjective Global Assessment (PG-SGA) was administered by trained clinical dietitians within 72 h of enrollment. According to established scoring criteria (18), patients were categorized into three groups: well-nourished (PG-SGA score 0–3), moderately malnourished or suspected malnutrition (score 4–8), and severely malnourished (score ≥9).

#### 2.3.5 Follow-up and outcome assessment

All patients were followed according to a protocol-defined schedule: every 3 months during the first 2 years post-treatment, every 6 months for the subsequent 3 years, and annually thereafter. Follow-up was conducted from April 2021 to April 2026. The primary endpoint of this study was overall survival (OS), defined as the time interval from study enrollment to death from any cause. Patients who were lost to follow-up were censored at the date of last contact. Survival status was verified through hospital records and telephone interviews.

### 2.4 Sample size calculation

Sample size was determined based on the primary endpoint of overall survival using the Schoenfeld formula for time-to-event data.

The required number of events (deaths) was calculated as:


$$d={\left(Z\alpha /2+Z\beta \right)}^{2}/[p(1-p)\times {\left(\ln\;\mathrm{HR}\right)}^{2}]$$

Where Zα/2 = 1.96 (two-sided α = 0.05), Zβ = 0.84 (β = 0.20, power = 80%), *p* = 0.5 (proportion of patients in the low phase angle group), and HR = hazard ratio comparing low versus high phase angle.

Based on a previous meta-analysis of phase angle in solid tumors reporting a hazard ratio of approximately 2.5 for low versus high phase angle (19), and our institutional data indicating that patients with stage IV disease (approximately 50% of our cohort) have a 5-year mortality rate of approximately 15–20%, the required number of events was calculated as:


$$\begin{array}{l} d={\left(1.96+0.84\right)}^{2}/[0.5\times 0.5\times {\left(\ln\;2.5\right)}^{2}]=\\ 7.84/(0.25\times 0.839)=7.84/0.210\approx 37\;\text{events} \end{array}$$

Accounting for an anticipated 15% event rate over 60 months (5 years) of follow-up (reflecting the 50% proportion of stage IV patients with poorer prognosis), the required total sample size was:


N=d/event rate=37/0.15≈247patients

To account for a potential 15% loss to follow-up and to ensure adequate statistical power for secondary analyses, the final sample size was set at 380 patients. With 27 observed deaths (7.1%) during the 60-month follow-up, the study achieves a reasonable number of events for the primary analysis given the favorable prognosis of newly diagnosed NPC patients. The events-per-variable ratio (EPV) in our multivariable model (3 variables, EPV = 9.0) approaches the recommended threshold of 10, supporting the reliability of the regression estimates.

### 2.5 Statistical analysis

Continuous variables were tested for normality using the Shapiro–Wilk test and presented as mean ± standard deviation (SD) or median with interquartile range (IQR), as appropriate. Group comparisons were performed using Student’s t-test, Mann–Whitney U test, chi-square test, or Fisher’s exact test, depending on variable distribution and type. All statistical analyses were conducted using R software (version 4.2.0) with the survival, survminer, timeROC, and pROC packages. Univariable Cox regression analyses were performed for each candidate prognostic factor. Variables with *p* < 0.10 in univariable analysis were considered for inclusion in the multivariable model. Given the number of events (*n* = 27), the multivariable model was restricted to include no more than three variables to avoid model overfitting. The proportional hazards assumption was formally tested for each covariate using Schoenfeld residuals; no significant violations were detected (global test *p* > 0.05 for all covariates). Two-tailed *p* < 0.05 was considered statistically significant, except for the univariable screening threshold (*p* < 0.10). Missing data accounted for less than 5% of all variables. Multiple imputation by chained equations was performed using the mice package in R (version 4.2.0), generating 20 imputed datasets. The imputation model included all variables listed in Table 1, as well as the event indicator and Nelson-Aalen estimator of cumulative hazard. Results were pooled using Rubin’s rules. Complete-case analysis yielded consistent results.

**Table 1:** Baseline characteristics of patients.

Characteristic	Total (*N* = 380)	Lower PA (< median) (*n* = 190)	Higher PA (≥ median) (*n* = 190)	*p*-value
Age (years), mean ± SD	54.187 ± 11.023	57.523 ± 10.512	50.876 ± 10.794	<0.001
Gender, *n* (%)				0.382
Male	247 (65.0)	125 (65.8)	122 (64.2)	
Female	133 (35.0)	65 (34.2)	68 (35.8)	
TNM stage, *n* (%)				0.023
III	190 (50.0)	85 (44.7)	105 (55.3)	
IV	190 (50.0)	105 (55.3)	85 (44.7)	
Phase angle (°), mean ± SD	4.81 ± 0.79	4.213 ± 0.27	5.624 ± 0.341	<0.001
BMI (kg/m^2^), mean ± SD	22.79 ± 2.90	21.76 ± 2.63	24.31 ± 2.68	<0.001
Albumin (g/L), mean ± SD	39.58 ± 4.51	37.58 ± 3.87	42.26 ± 3.89	<0.001
PG-SGA score, mean ± SD	9.312 ± 2.80	10.62 ± 2.48	7.394 ± 2.01	<0.001
PG-SGA category, *n* (%)				<0.001
Well-nourished (0–6)	124 (32.6)	42 (22.1)	82 (43.2)	
Moderate malnutrition (7–11)	172 (45.3)	86 (45.3)	86 (45.3)	
Severe malnutrition (≥12)	84 (22.1)	62 (32.6)	22 (11.6)	
Treatment, *n* (%)				0.628
CCRT alone	304 (80.0)	152 (80.0)	152 (80.0)	
IC + CCRT	76 (20.0)	38 (20.0)	38 (20.0)	
Smoking status, *n* (%)				0.315
Never	178 (46.8)	88 (46.3)	90 (47.4)	
Former/current	202 (53.2)	102 (53.7)	100 (52.6)	
Alcohol consumption, *n* (%)				0.482
No	242 (63.7)	124 (65.3)	118 (62.1)	
Yes	138 (36.3)	66 (34.7)	72 (37.9)	

## 3 Results

### 3.1 Patient characteristics

A total of 380 patients with histologically confirmed newly diagnosed nasopharyngeal carcinoma (NPC) who underwent chemoradiotherapy were prospectively enrolled in this cohort study. The mean phase angle was 4.812° ± 0.792°. For descriptive purposes, patients were stratified by the median PA value of the cohort. Table 1 summarizes the baseline demographic and clinical characteristics of the study population according to this descriptive stratification.

Significant differences were observed between the two groups across multiple parameters. Patients in the lower PA group were significantly older than those in the higher PA group (57.52 ± 10.51 years vs. 50.87 ± 10.79 years, *p* < 0.001). Among patients, those with lower PA presented with more advanced disease, as evidenced by a higher proportion of stage IV disease (55.3% vs. 44.7%, *p* = 0.023).

All conventional nutritional indicators consistently demonstrated poorer nutritional status in patients with lower PA. Specifically, these patients exhibited significantly lower BMI (21.76 ± 2.63 kg/m^2^ vs. 24.31 ± 2.68 kg/m^2^, *p* < 0.001), lower serum albumin levels (37.58 ± 3.87 g/L vs. 42.26 ± 3.89 g/L, p < 0.001), and higher PG-SGA scores (10.62 ± 2.48 vs. 7.39 ± 2.01, p < 0.001).

In contrast, no statistically significant differences were observed between the two groups regarding sex distribution (male: 65.8% vs. 64.2%, *p* = 0.382), treatment modality, smoking status, or alcohol consumption.

### 3.2 Survival outcomes

**Figure 1:**
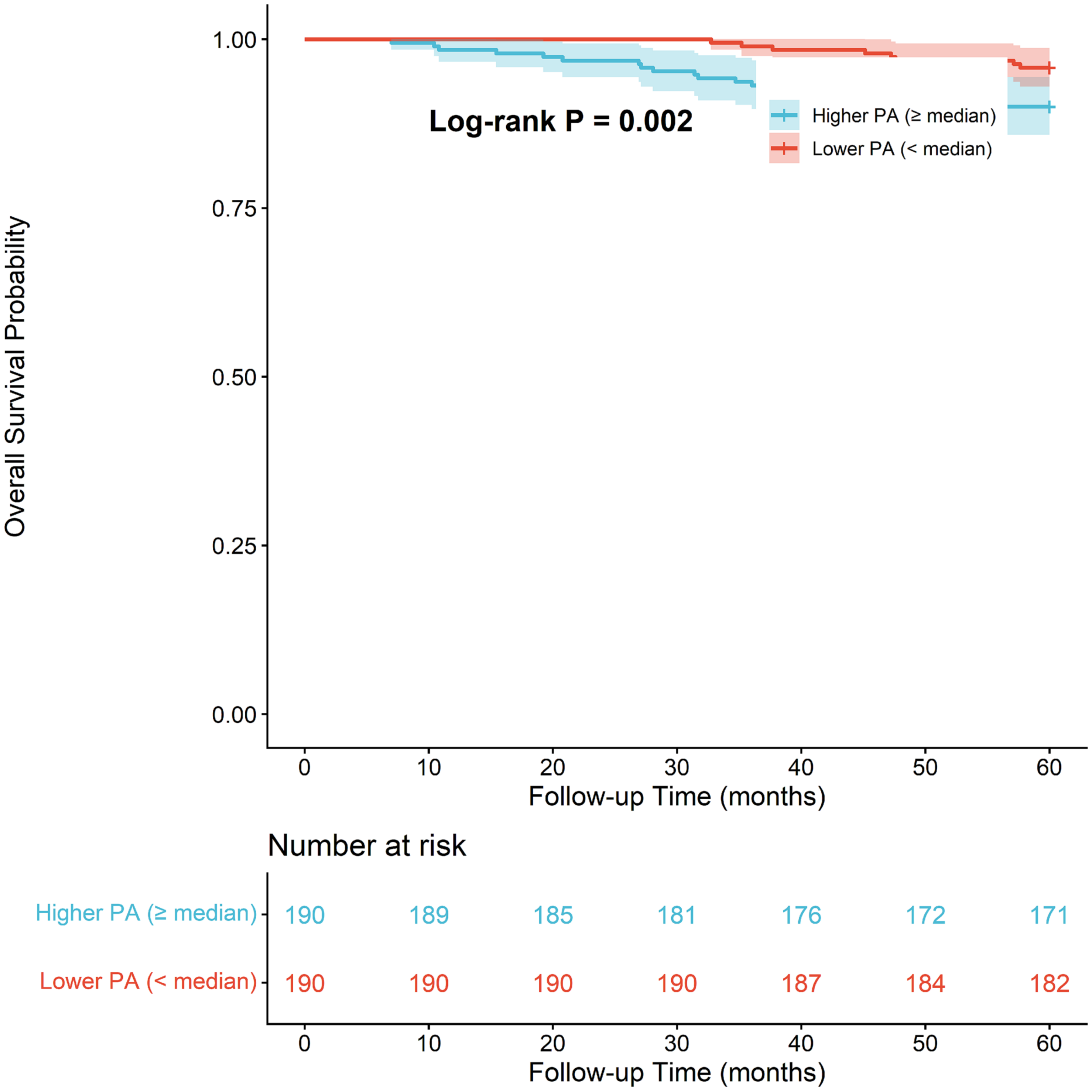
Kaplan–Meier survival curves for overall survival according to phase angle.

During a median follow-up period of 60 months (range: 6 to 60 months), 27 deaths (7.1%) were documented among the 380 newly diagnosed NPC patients. Kaplan–Meier survival analysis using median-based stratification for visualization demonstrated that patients in the lower PA group experienced significantly worse overall survival compared to those in the higher PA group (log-rank *p* = 0.002; Figure 1). The 60 -month survival rates were 86.8% (95% CI: 82.156–91.523) in the lower PA group versus 93.7% (95% CI: 90.184–97.267) in the higher PA group.

### 3.3 Correlation between phase angle and conventional nutritional indicators

**Figure 2:**
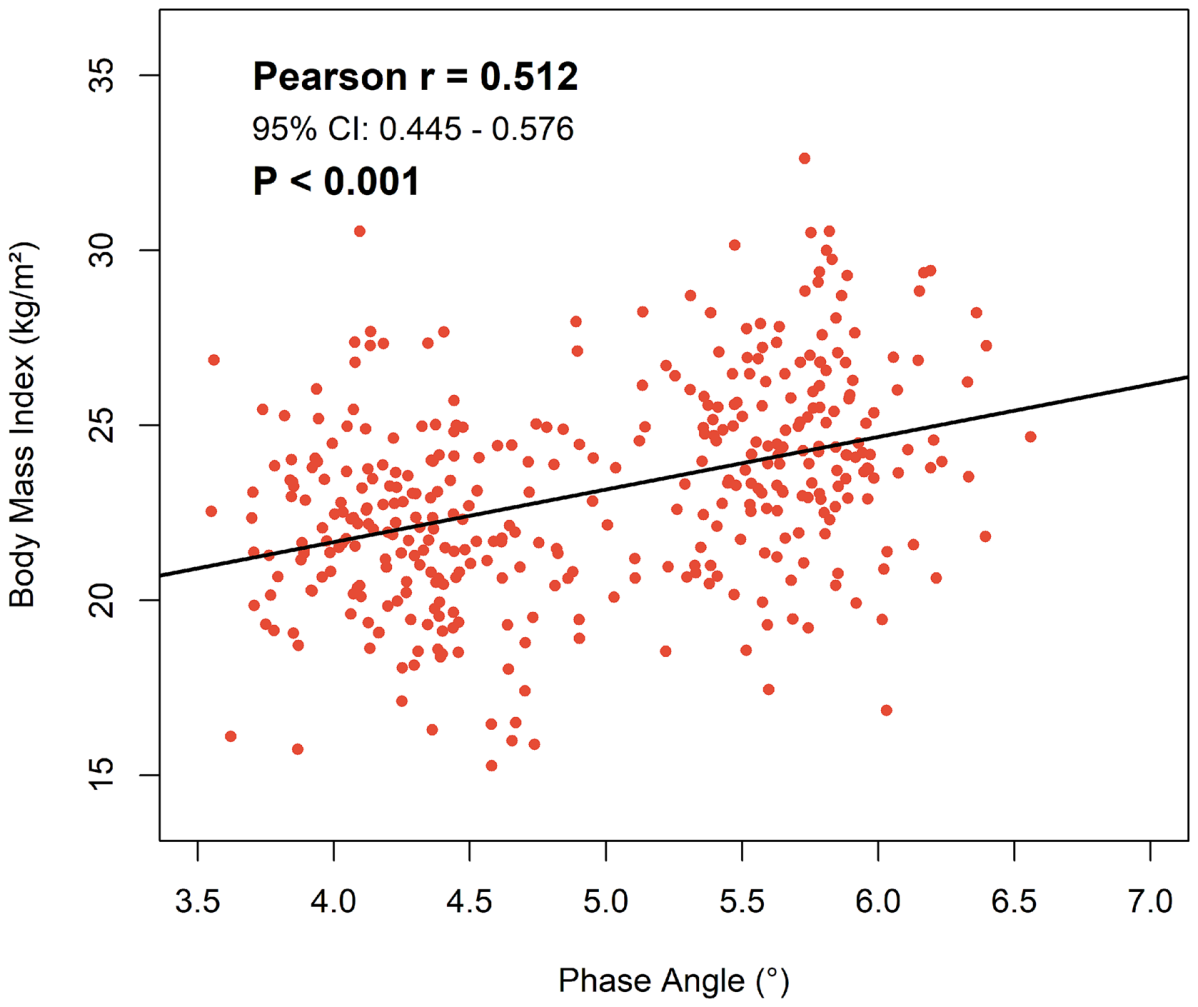
Correlation between phase angle and body mass index (BMI).

**Figure 3:**
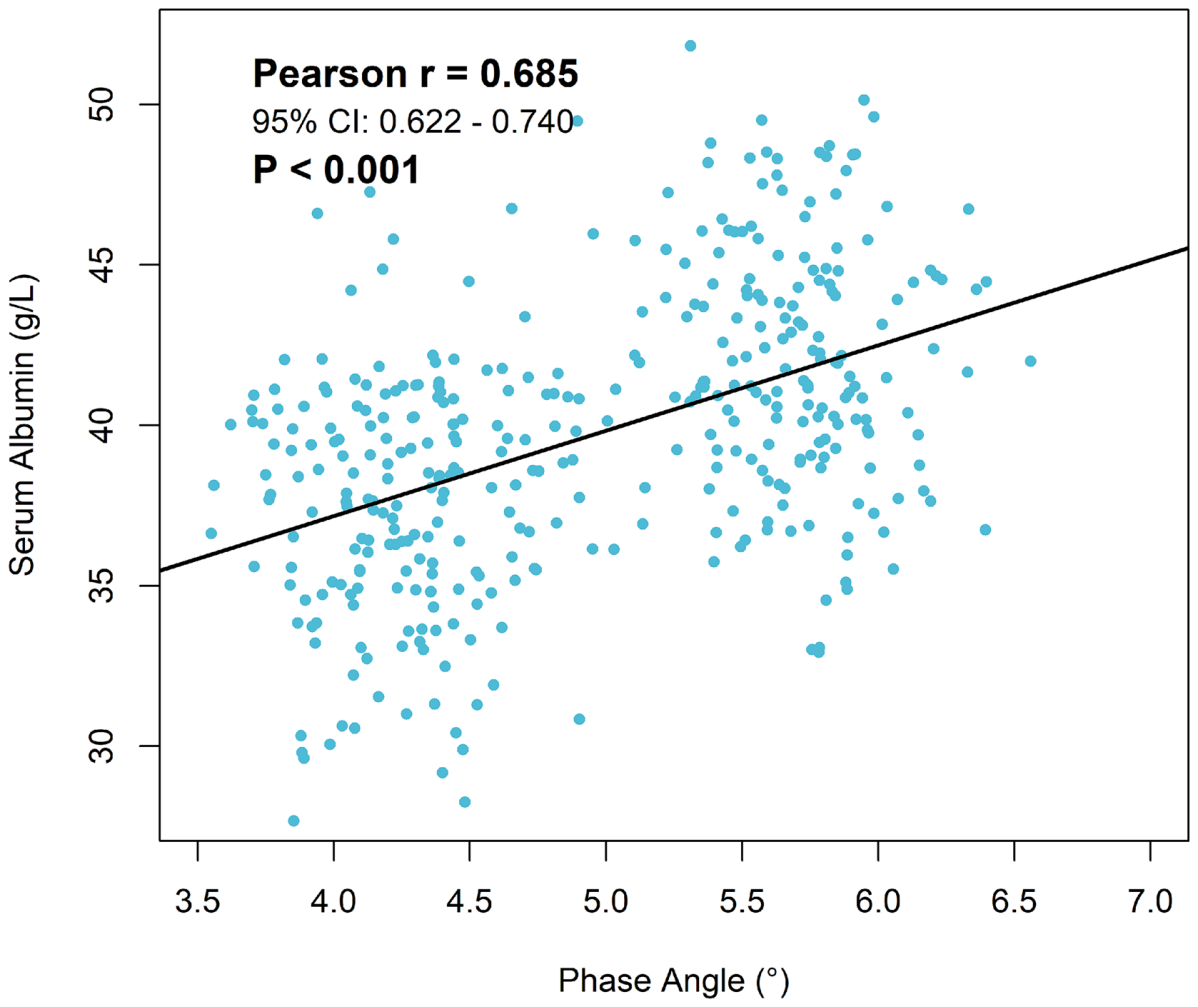
Correlation between phase angle and serum albumin.

**Figure 4:**
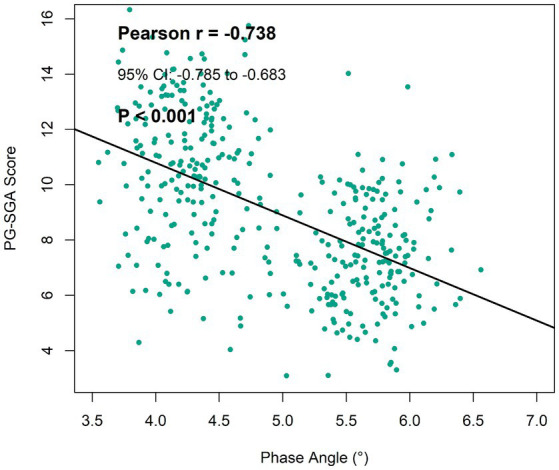
Correlation between phase angle and Patient-Generated Subjective Global Assessment (PG-SGA) score.

Phase angle showed a moderate positive correlation with BMI (Pearson r = 0.512, 95% CI: 0.445–0.576, *p* < 0.001; Figure 2). A strong positive correlation was observed between phase angle and serum albumin (r = 0.685, 95% CI: 0.622–0.740, p < 0.001; Figure 3). Conversely, phase angle demonstrated a strong negative correlation with PG-SGA score (r = −0.738, 95% CI: −0.785 to −0.683, p < 0.001; Figure 4).

### 3.4 Univariable and multivariable cox regression analysis

**Figure 5:**
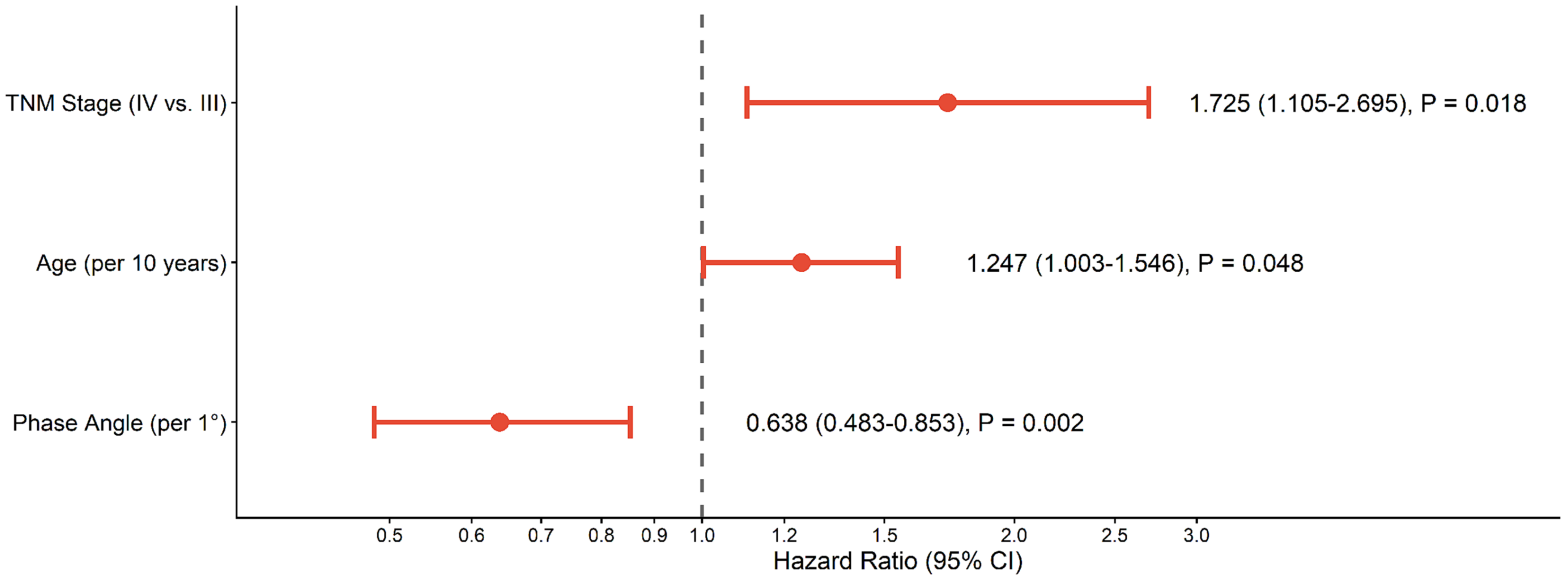
Forest plot of multivariable Cox regression analysis for overall survival.

Univariable Cox regression analyses identified phase angle, age, TNM stage, BMI, albumin, and PG-SGA as significant predictors of overall survival (all *p* < 0.05; Table 2). In multivariable Cox regression analysis adjusting for age and TNM stage (3-variable model, EPV = 9.0), phase angle remained an independent predictor of overall survival. Each 1° increase in phase angle was associated with a 35.8% reduction in mortality risk (HR = 0.642, 95% CI: 0.485–0.850, *p* = 0.002; Table 2; Figure 5). Age (HR = 1.242 per 10-year increase, 95% CI: 1.002–1.542, *p* = 0.048) and TNM stage (HR = 1.722 for stage IV vs. III, 95% CI: 1.102–2.692, *p* = 0.018) also remained significant independent predictors in the multivariable model (Table 2; Figure 5).

**Table 2:** Univariable and Multivariable Cox Regression Analysis for Overall Survival.

Variable	Univariable HR (95% CI)	*P*-value	Multivariable HR (95% CI)	*P*-value
Phase angle (per 1° increase)	0.587 (0.441–0.769)	<0.001	0.638 (0.483–0.853)	0.002
Age (per 10-year increase)	1.315 (1.058–1.628)	0.012	1.247 (1.003–1.546)	0.048
TNM stage (IV vs. III)	1.978 (1.279–3.068)	0.002	1.725 (1.105–2.695)	0.018
BMI (per 1 kg/m^2^ increase)	0.925 (0.863–0.987)	0.012	—	—
Albumin (per 1 g/L increase)	0.945 (0.894–0.996)	0.021	—	—
PG-SGA (per 1-point increase)	1.156 (1.064–1.257)	0.001	—	—

Multivariable model adjusted for phase angle (continuous), age, and TNM stage (3 variables, EPV = 9.0). BMI, albumin, and PG-SGA were not included due to high correlation with phase angle and to preserve degrees of freedom.

### 3.5 Comparison of predictive accuracy

**Figure 6:**
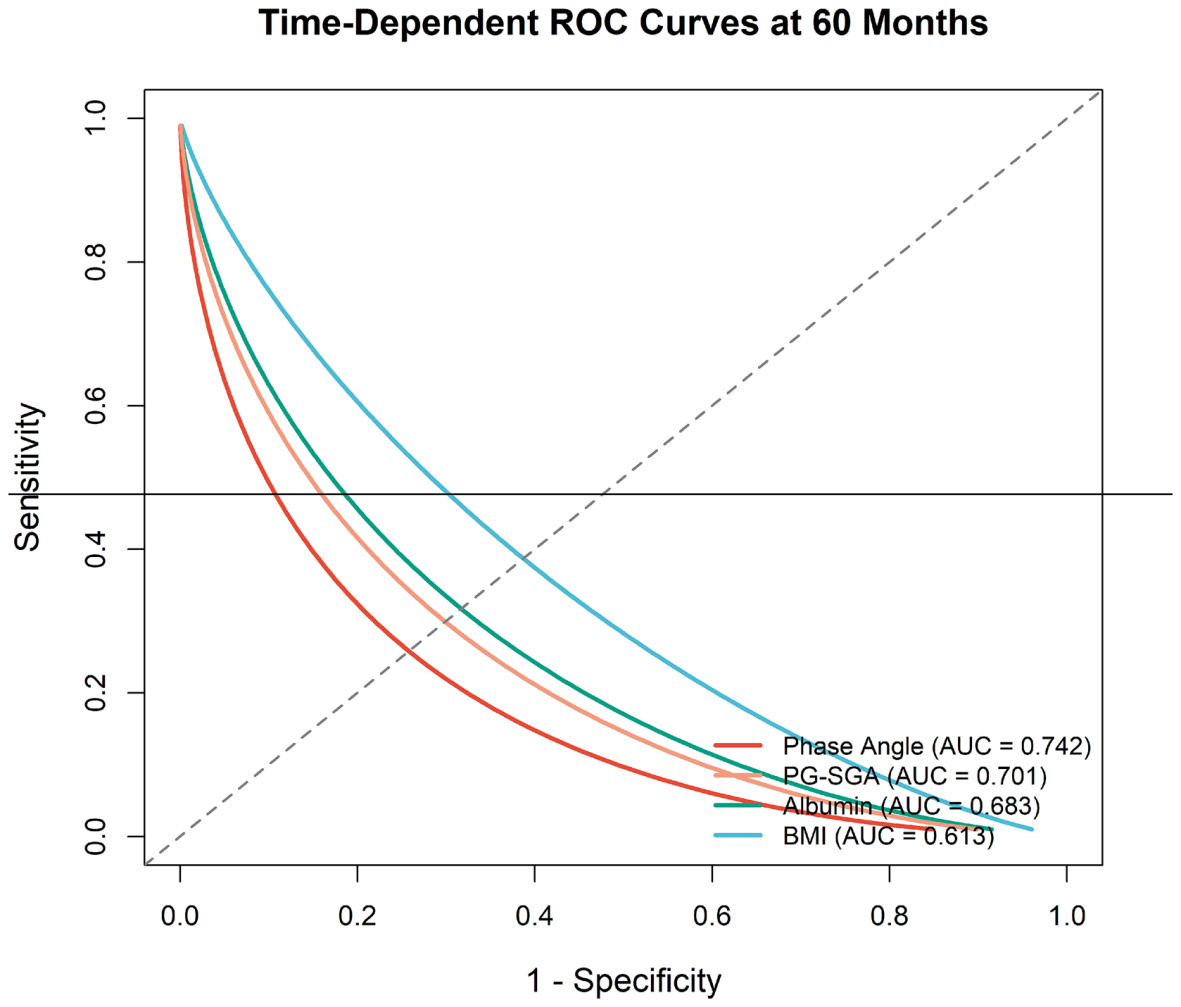
Time-dependent receiver operating characteristic (ROC) curves for phase angle and conventional nutritional indicators at 60 months.

Phase angle (as a continuous variable) demonstrated an AUC of 0.742 (95% CI: 0.655–0.829), compared to 0.613 (95% CI: 0.523–0.703) for BMI, 0.683 (95% CI: 0.593–0.773) for albumin, and 0.701 (95% CI: 0.611–0.791) for PG-SGA (Table 3; Figure 6).

**Table 3:** Predictive accuracy of phase angle and conventional nutritional indicators.

Indicator	AUC (95% CI)	Sensitivity	Specificity	Youden Index
Phase angle (continuous)	0.742 (0.655–0.829)	0.731	0.682	0.413
BMI	0.613 (0.523–0.703)	0.582	0.601	0.183
Albumin	0.683 (0.593–0.773)	0.662	0.634	0.296
PG-SGA	0.701 (0.611–0.791)	0.684	0.642	0.326

## 4 Discussion

This prospective cohort study provides, to our knowledge, the first head-to-head comparison of phase angle with conventional nutritional indicators in newly diagnosed nasopharyngeal carcinoma (NPC) patients undergoing chemoradiotherapy. Our findings suggest that pre-treatment PA may independently predict overall survival and shows promising prognostic accuracy compared to conventional indicators over a 60-month follow-up period. However, given the moderate number of events (n = 27) and the single-center nature of this study, these results should be interpreted with caution and considered hypothesis-generating rather than definitive.

Comparison with Previous Studies.

The prognostic significance of phase angle observed in our NPC cohort aligns with findings from studies across diverse malignancies. In gastrointestinal cancer patients, phase angle has been independently associated with survival outcomes and demonstrated superior prognostic performance compared to traditional nutritional markers (20). Similarly, studies in lung cancer have reported that reduced phase angle predicts poor prognosis and correlates with diminished quality of life (17). A meta-analysis encompassing 30 studies with 6,587 cancer patients confirmed that a higher phase angle was significantly associated with improved overall survival (pooled HR = 0.73, 95% CI: 0.66–0.81, *p* < 0.001), indicating that patients with low phase angle values had a 27% lower probability of survival compared to those with high values (21). Our study extends this evidence to nasopharyngeal carcinoma—a head and neck cancer with unique treatment-related nutritional challenges, including radiation-induced oral mucositis, dysphagia, odynophagia, and xerostomia that collectively impair nutritional intake and exacerbate malnutrition during treatment (22).

Notably, our direct comparison between phase angle and conventional nutritional indicators revealed that phase angle predicted survival independently of age and TNM stage.

### 4.1 Biological plausibility

Several interconnected biological mechanisms may explain the superior prognostic value of phase angle (23, 24). Fundamentally, phase angle serves as a direct biophysical measure of cellular membrane integrity and function, derived from the relationship between resistance (reflecting extracellular and intracellular fluid volumes) and reactance (reflecting cell membrane capacitance and cellular health) (12, 13). A reduced phase angle signifies compromised cellular integrity, increased apoptosis, heightened inflammatory activity, and depletion of body cell mass (14).

In NPC patients undergoing chemoradiotherapy, multiple pathological processes converge to influence phase angle. Tumor burden and treatment-related toxicities induce oxidative stress and systemic inflammation, characterized by elevated pro-inflammatory cytokines such as interleukin-6 and tumor necrosis factor-*α* (25). These inflammatory mediators promote cellular damage, alter membrane permeability, and disrupt ionic gradients—all of which are captured by phase angle measurements (26). Furthermore, radiation-induced mucosal injury activates local and systemic inflammatory responses, potentially exacerbating cellular dysfunction and further reducing phase angle values (27, 28).

An important advantage of phase angle over conventional nutritional indicators lies in its integrative nature. Unlike BMI, which provides only a crude anthropometric measure, phase angle incorporates information about both the quantity and quality of cellular mass. Unlike albumin, which is confounded by inflammation, phase angle directly reflects the cellular consequences of inflammatory processes. Unlike PG-SGA, which relies on subjective patient reporting, phase angle provides an objective, reproducible biophysical measurement. These characteristics may explain why phase angle retained prognostic significance in multivariable models where conventional indices were attenuated to non-significance.

### 4.2 Clinical implications

Our findings carry several clinical implications. First, the non-invasive, rapid, and inexpensive nature of phase angle measurement makes it suitable for integration into routine clinical practice. BIA devices are increasingly available in oncology settings, and measurements require minimal patient burden and can be completed within minutes during outpatient visits. Second, we recommend that phase angle be interpreted as a continuous variable in clinical practice, with higher values indicating better prognosis. Third, given the moderate predictive accuracy (AUC = 0.742), phase angle should be considered a complementary tool to conventional nutritional assessment rather than a replacement. It may help identify high-risk patients who might benefit from individualized nutritional interventions before and during chemoradiotherapy, but this requires confirmation in prospective interventional studies.

### 4.3 Strengths

This study has several strengths. First, the prospective cohort design with standardized BIA measurement protocols enhances data quality and reproducibility. Second, we excluded patients with recurrent or metastatic disease, reducing heterogeneity and confounding related to treatment intent and prognosis. Third, we formally tested the proportional hazards assumption using Schoenfeld residuals and reported detailed multiple imputation parameters (20 imputed datasets) to enhance methodological transparency.

### 4.4 Limitations

Several limitations of this study should be acknowledged.

First, and most importantly, while 27 events provide a reasonable basis for the primary analysis, the events-per-variable ratio (EPV = 9.0 with 3 variables) is slightly below the recommended threshold of 10. This suggests that the regression estimates, while statistically significant, should be interpreted with caution and may be subject to some degree of overfitting.

Second, the moderate event number also results in wide confidence intervals for AUC estimates. Consequently, pairwise comparisons of AUCs using DeLong’s test were not performed, as they would be statistically underpowered. The AUC values are presented descriptively for exploratory purposes only.

Third, this was a single-center study conducted in Southern China, which may limit generalizability to other populations and healthcare settings with different ethnic compositions and treatment protocols.

Fourth, although we excluded patients with recurrent/metastatic disease, the inclusion of both stage III and IV patients introduces some heterogeneity in baseline prognosis.

Fifth, phase angle was assessed only at baseline; serial measurements during and after treatment might provide additional prognostic information regarding dynamic changes in nutritional status and cellular health.

Sixth, detailed body composition parameters (e.g., lean mass, fat-free mass, sarcopenia indices) were not collected, limiting our ability to fully characterize the relationship between phase angle and body composition abnormalities common in NPC patients.

Seventh, no calibration analysis, clinical decision curve analysis, or comparison with combined predictive models was performed, which would be important to fully demonstrate the incremental value of phase angle over traditional markers. Future research should address these aspects.

Given these limitations, our findings should be considered exploratory and hypothesis-generating, and validation in larger multicenter cohorts with extended follow-up and adequate statistical power is warranted before routine clinical implementation can be recommended.

### 4.5 Future directions

Building on our findings, several research priorities emerge. First, multicenter prospective studies with diverse geographic and ethnic representation are needed to validate the prognostic value of phase angle and establish population-specific reference values. Second, mechanistic studies incorporating serial measurements of inflammatory markers and detailed body composition analysis could elucidate the biological pathways linking phase angle to survival. Third, randomized controlled trials should test whether phase angle-guided nutritional interventions can improve clinically meaningful outcomes in high-risk patients. Finally, future studies should explore whether serial phase angle measurements during treatment provide dynamic prognostic information superior to a single baseline assessment.

## Data Availability

The raw data supporting the conclusions of this article will be made available by the authors, without undue reservation.

## References

[1] Chen YP Chan ATC Le QT Blanchard P Sun Y Ma J . Nasopharyngeal carcinoma. Lancet. (2019) 394:64–80. doi: 10.1016/j.oraloncology.2011.06.057 31178151

[2] Tang LL Guo R Zhang N Deng B Chen L Cheng Z-B . Effect of radiotherapy alone vs radiotherapy with concurrent chemoradiotherapy on survival without disease relapse in patients with low-risk nasopharyngeal carcinoma: a randomized clinical trial. JAMA. (2022) 328:728–36. doi: 10.1001/jama.2022.13997, 35997729 PMC9399866

[3] Miao J Wang L Ong EHW Hu C Lin S Chen X . Effects of induction chemotherapy on nutrition status in locally advanced nasopharyngeal carcinoma: a multicentre prospective study. J Cachexia Sarcopenia Muscle. (2023) 14:815–25. doi: 10.1002/jcsm.13196 36872457 PMC10067484

[4] Miao J Wang L Hu C Lin S Tan SH Ong E . A multicenter prospective observational study of nutritional status on survival in locally advanced nasopharynx cancer treated by induction chemotherapy and chemoradiotherapy. J Clin Oncol. (2019) 37:6036–6. doi: 10.1200/JCO.2019.37.15_suppl.6036

[5] Wang P Soh KL Ying Y Liao J Huang X Zhao H . Risk factors for malnutrition in patients with nasopharyngeal carcinoma. Support Care Cancer. (2023) 31:723. doi: 10.1007/s00520-023-08166-8, 38008866

[6] Zhou K Gou X Ma H He PG Tian X Xu H. Analysis of nutritional status of nasopharyngeal carcinoma patients and its impact on quality of life. Parent Enteral Nutr. (2024) 31:280–7. doi: 10.16151/j.1007-810x.2024.05.005

[7] Li Y Feng H Hu J Fan XY Chen XW. Application of PG-SGA and GLIM in nutritional assessment of inpatients with nasopharyngeal carcinoma. Acad J Guangzhou Med Univ. (2025) 53:46–50. doi: 10.3969/j.issn.2095-9664.2025.05.07

[8] Luo B He D Jiang X Li ZL He LL Wu XL. Prognostic value of nutritional index, body mass index and N stage before chemoradiotherapy in prognostic prediction of nasopharyngeal carcinoma patients. Electr J Metab Nutri Cancer. (2023) 10:786–92. doi: 10.16689/j.cnki.cn11-9349/r.2023.06.014

[9] Jia P Wu X Shen F Xu G Xu H Cong M . Nutritional status and its correlation to prognosis of nasopharyngeal carcinoma patients in different ages in China: a multicenter cohort study. Support Care Cancer. (2023) 31:638. doi: 10.1007/s00520-023-08104-8, 37847417

[10] Yang Y Yuying Zhao Y. A consistency study of global leadership initiative on malnutrition standard patient-generated subjective global assessment and serum albumin in diagnosis of malnutrition in cancer patients. J Clin Med Pract. (2022) 26:82–7. doi: 10.7619/jcmp.20212940

[11] Arab A Karimi E Vingrys K Shirani F. Is phase angle a valuable prognostic tool in cancer patients' survival? A systematic review and meta-analysis of available literature. Clin Nutr. (2021) 40:3182–90. doi: 10.1016/j.clnu.2021.01.027, 33581951

[12] Bellido D García-García C Talluri A Lukaski HC García-Almeida JM. Future lines of research on phase angle: strengths and limitations. Rev Endocr Metab Disord. (2023) 24:563–83. doi: 10.1007/s11154-023-09803-7, 37043140 PMC10090740

[13] Norman K Stobäus N Pirlich M Bosy-Westphal A. Bioelectrical phase angle and impedance vector analysis--clinical relevance and applicability of impedance parameters. Clin Nutr. (2012) 31:854–61. doi: 10.1016/J.CLNU.2012.05.008 22698802

[14] Lukaski HC Talluri A. Phase angle as an index of physiological status: validating bioelectrical assessments of hydration and cell mass in health and disease. Rev Endocr Metab Disord. (2023) 24:371–9. doi: 10.1007/s11154-022-09764-3, 36336754

[15] Amano K Bruera E Hui D. Diagnostic and prognostic utility of phase angle in patients with cancer. Rev Endocr Metab Disord. (2023) 24:479–89. doi: 10.1007/s11154-022-09776-z, 36484944

[16] Boćkowska M Kostro P Kamocki ZK. Phase angle and postoperative complications in a model of Immunonutrition in patients with pancreatic Cancer. Nutrients. (2023) 15:328. doi: 10.3390/nu15204328, 37892404 PMC10609395

[17] Detopoulou P Voulgaridou G Papadopoulou S. Cancer phase angle and sarcopenia: the role of diet in connection with lung cancer prognosis. Lung. (2022) 200:347–79. doi: 10.1007/s00408-022-00536-z, 35616720

[18] Da Silva BR Kirkham AA Ford KL Haykowsky MJ Paterson DI Joy AA . Phase angle is associated with muscle health and cardiorespiratory fitness in older breast cancer survivors. Clin Nutr ESPEN. (2023) 55:37202048. doi: 10.1016/j.clnesp.2023.03.019, 37202048

[19] Shi YY Zhang XW Yuan KT Xue CL Yu HL Shi HP. Introduction to PG-SGA operation standards. Chin J Cancer Prev Treat. (2013) 20:1779–82. doi: 10.3969/j.issn.1673-5269.2013

[20] Popiolek-Kalisz J Kalisz G. Malnutrition assessed with phase angle and mortality risk in heart failure - a meta-analysis. Nutr Metab Cardiovasc Dis. (2025) 35:104222. doi: 10.1016/j.numecd.2025.104222, 40887361

[21] Baş D Atahan C Tezcanli E. An analysis of phase angle and standard phase angle cut-off values and their association with survival in head and neck cancer patients undergoing radiotherapy. Clin Nutr. (2023) 42:1445–53. doi: 10.1016/j.clnu.2023.06.020, 37451156

[22] Yang J Xie H Wei L Ruan G Zhang H Shi J . Phase angle: a robust predictor of malnutrition and poor prognosis in gastrointestinal cancer. Nutrition. (2024) 125:112468. doi: 10.1016/j.nut.2024.112468, 38781749

[23] Kong Q Tian L Wang Y Yu M. Phase angle as a prognostic factor in patients with cancer: a systematic review of the existing evidence via a meta-analysis. Nutr Hosp. (2025) 42:161–72. doi: 10.37766/inplasy2024.10.0025 39692239

[24] Sat-Muñoz D Martínez-Herrera BE González-Rodríguez JA Gutiérrez-Rodríguez LX Trujillo-Hernández B Quiroga-Morales LA . Phase angle, a cornerstone of outcome in head and neck Cancer. Nutrients. (2022) 14. doi: 10.3390/nu14153030, 35893884 PMC9330539

[25] Di Vincenzo O Marra M Di Gregorio A Pasanisi F Scalfi L. Bioelectrical impedance analysis (BIA) -derived phase angle in sarcopenia: a systematic review. Clin Nutr. (2021) 40:33183880:3052–61. doi: 10.1016/j.clnu.2020.10.048 33183880

[26] Rinaldi S Gilliland J O'Connor C Chesworth B Madill J. Is phase angle an appropriate indicator of malnutrition in different disease states? A systematic review. Clin Nutr. (2019) 29:1–14. doi: 10.1016/j.clnesp.2018.10.010, 30661671

[27] Li D Fan G Zhou Y. Chitinase 3 like-1 activates the Akt pathway, inducing NF-κB-dependent release of pro-inflammatory cytokines and promoting the proliferative ability in nasopharyngeal carcinoma cells. Cytokine. (2024) 179:156631. doi: 10.1016/j.cyto.2024.156631, 38710115

[28] Zhou S Yu Z Shi X Zhao H Dai M Chen W. The relationship between phase angle, nutrition status, and complications in patients with pancreatic head Cancer. Int J Environ Res Public Health. (2022) 19:6426. doi: 10.3390/ijerph19116426, 35682009 PMC9180801

